# Mechanical Feedback Control for Multicellular Tissue Size Maintenance: A Minireview

**DOI:** 10.3389/fcell.2021.820391

**Published:** 2022-01-14

**Authors:** Tsuyoshi Hirashima

**Affiliations:** ^1^ The Hakubi Center, Kyoto University, Kyoto, Japan; ^2^ Laboratory of Bioimaging and Cell Signaling, Graduate School of Biostudies, Kyoto University, Kyoto, Japan; ^3^ Japan Science and Technology Agency, PRESTO, Kawaguchi, Japan

**Keywords:** epithelial tissues, control system, feedback regulation, mechano-response, tissue size maintenance

## Abstract

All living tissues and organs have their respective sizes, critical to various biological functions, such as development, growth, and homeostasis. As tissues and organs generally converge to a certain size, intrinsic regulatory mechanisms may be involved in the maintenance of size regulation. In recent years, important findings regarding size regulation have been obtained from diverse disciplines at the molecular and cellular levels. Here, I briefly review the size regulation of biological tissues from the perspective of control systems. This minireview focuses on how feedback systems engage in tissue size maintenance through the mechanical interactions of constituent cell collectives through intracellular signaling. I introduce a general framework of a feedback control system for tissue size regulation, followed by two examples: maintenance of epithelial tissue volume and epithelial tube diameter. The examples deliver the idea of how cellular mechano-response works for maintaining tissue size.

## Introduction

The size of biological tissues and organs is a crucial variable closely related to various biological functions. Researchers have long been fascinated with biological size control since the pioneering essay by John Haldane, “On being the right size” in 1926 (Haldane, 1926). “How do organs know when they have reached the right size?” (Travis, 2013) is an old question that has remained unanswered till date. In recent years, extensive research has focused on understanding tissue size control as a system, in which the individual cells that make up the tissue interact with each other (Lander, 2011; Penzo-Mendez and Stanger, 2015; Boulan and Léopold, 2021). In this minireview, I describe how multicellular tissue size is regulated considering the control system in the context of mechanobiology, wherein the size is defined according to the dimension of interest as the target characteristic. In other words, size does not necessarily mean volume; it can correspond to the length or area in some cases. For example, when discussing the size of tubes, a typical structure of epithelial tissues, we often focus on their longitudinal or cross-sectional diameter, as discussed in a later section. The impact of the extracellular matrix on tissue size regulation has not been discussed, although it is critical in some cases.

## Feedback Control System for Tissue and Organ Size

**FIGURE 1:**
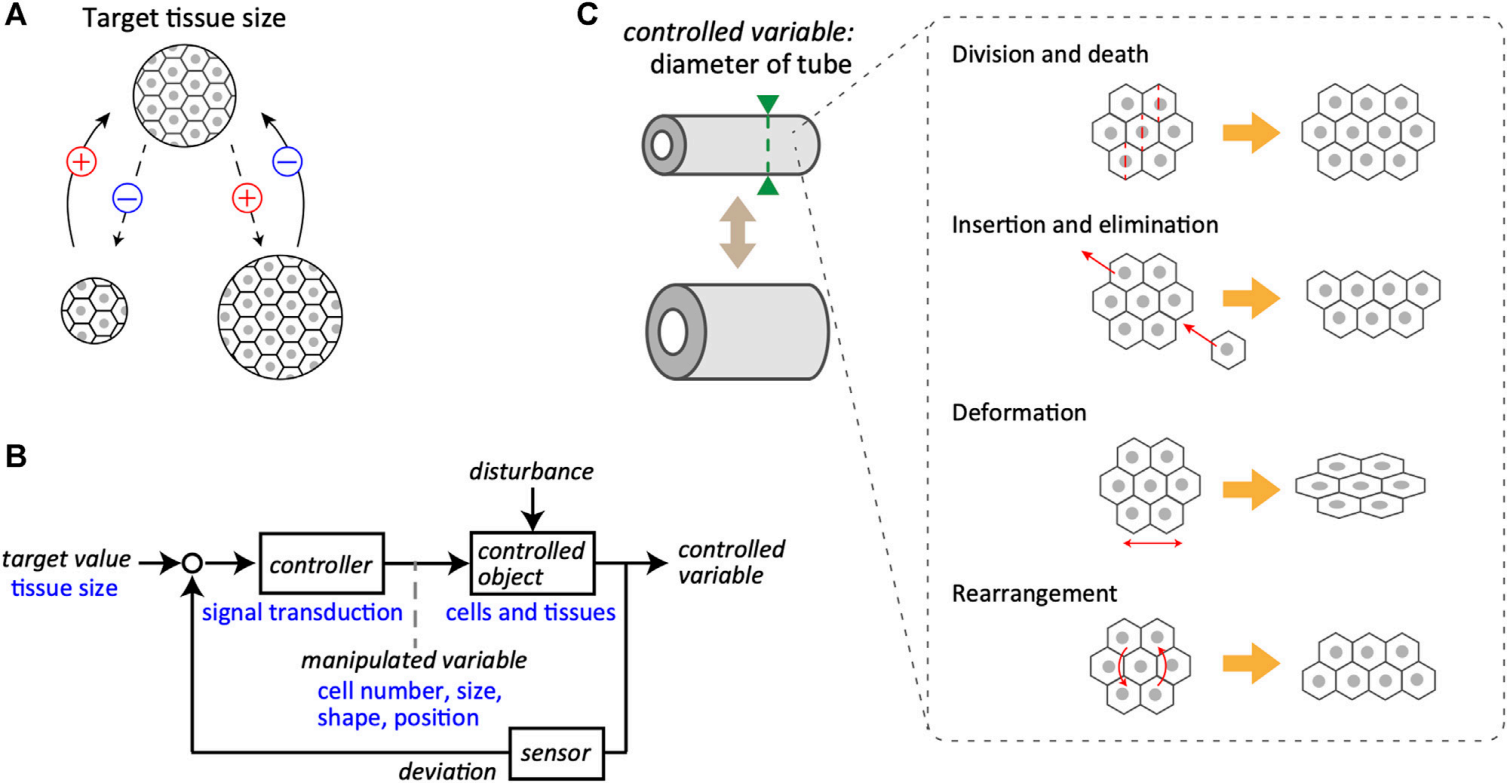
Feedback control system in tissue size maintenance. **(A)** Schematics of the negative feedback loop for the maintenance of tissue size. A specific size of tissue, composed of cells, is set as the target value (top). Even if the tissue size may change during biological processes (bottom), it returns to the target value due to the negative feedback regulation. The plus and minus signs represent the notions of positive and negative change in the tissue size, respectively. **(B)** A block diagram of the control system for tissue size maintenance. General terms used in system engineering are indicated in black, whereas the corresponding examples of biological objects are in blue. Target value of tissue size is maintained by the closed loop mediated through controller, controlled object, and sensor. Note that the manipulated variable bridges from controller to controlled object. **(C)** Examples of manipulated variables for tube diameter as the controlled variable, including cell division and death, local cell insertion and elimination, cell deformation, and cell rearrangement.

The issue of size regulation can be divided into two main classes: determination and maintenance of the right size. This section focuses on a control system that maintains the target value of the right size, provided that it has been determined *via* certain mechanisms. In most typical cases, the size of any living tissue does not diverge over time and is maintained at a specific value. This suggests the presence of a negative feedback loop, which is indispensable for the homeostasis and stabilization of the system, at the core of the regulatory mechanisms (Figure 1A). In other words, biological tissues are equipped with a system in which the difference between the current and target values is calculated, allowing the system to decrease and increase the size depending on the positive and negative differences, respectively.

Regarding the basic structure of the feedback control system that regulates tissue size, the system considered here is a closed-loop system for converging the size of tissues and organs to the target value (Figure 1B). Individual cells in the tissues receive information about the current size value through sensors and calculate the deviation from the target value. To reduce deviation, the cells yield manipulated variables, that is, output behaviors, such as the cell size, shape, and positions. This regulation proceeds through intracellular and intercellular signal transductions that act as the controller. When the manipulated quantity is supplemented to the controlled objects, such as cells and tissues, a controlled variable is updated. The controlled object is generally subjected to external disturbances along with the manipulated variables inside the system. A clear example of a disturbance is the partial resection of an organ. The mammalian liver can be considered an example of regenerative ability; in rats, it is known to recover its original size and function even after two-thirds of the liver is removed (Higgins, 1931; Taub, 2004). This suggests that certain organs possess mechanisms to robustly maintain their size against a large degree of disturbance and that the actual size is controlled by a coordinated coupling of each component in the feedback system, including target value, controller, controlled object, and sensor. The feedback loop is an essential control regulatory network for the size regulation of biological tissues, subjected to unpredictable disturbances.

In the following subsections, I provide brief descriptions of each component of the feedback control system concerning biological events.

### Target Value

The specific size of tissues is the target or desired value for the system and is determined by various factors, including genetic and physical constraints. In addition, tissue size varies according to life events, such as development, growth, and disease. For example, the number of pancreatic beta cells in pregnancy and obesity increases several times compared to that under normal conditions. This is because pancreatic beta cells, or insulin-producing cells, proliferate to compensate for increased insulin resistance, thereby preventing hyperglycemia in diabetes (Zhou and Melton, 2018). There are also cases where the target size value increases locally in tissues because of endocrine or metabolic abnormalities, as in the case of the local gigantism of fingers and toes, also known as macrodactyly (Kalen et al., 1988).

### Controller

The controller is a core function that operates a system and corresponds to the device connecting the input and output, namely the signal transduction system of the cells. It receives information on tissue size as an input through chemical factors or mechanical forces caused by the tension between neighboring cells. In either case, the molecules and signaling pathways involved in regulating tissue size have been intensively investigated (Boulan et al., 2015). In particular, the Hippo signaling pathway has been well studied in this context (Yu et al., 2015; Panciera et al., 2017). In the downstream of the Hippo pathway, Yes-associated protein (YAP) and transcriptional co-activator with PDZ-binding motif (TAZ) regulate gene expressions with transcriptional factors TEADs, controlling the cell proliferation. Active YAP subcellular localization is more evident in the nucleus compared in the cytoplasm under higher cell densities, indicating the Hippo-YAP/TAZ signaling contributes to the tissue volume maintenance in response to mechanical tension within the cells and/or cell morphology (Dupont et al., 2011; Wada et al., 2011).

### Manipulated Variable

As the target value is related to tissue volume, the origins of the manipulated variables include various cellular behaviors related to the net change in volume, such as cell proliferation, hypertrophy, death, and atrophy. Epithelial-mesenchymal transition is also involved, as it corresponds to cell insertion and elimination in the tissue of interest. These cellular behaviors are directly linked to changes in tissue volume. However, if the target value is related to the cross-sectional diameter of the multicellular tube, the cellular behaviors as manipulated variables are more diverse (Figure 1C). For example, in proliferating monolayer epithelial tubes, the orientation of cell division is biased to the longitudinal axis, and the volume increase due to cell proliferation would be reflected mostly in the extension of the longitudinal axis but not of the circumferential axis of the tubes (Gillies and Cabernard, 2011; Tang et al., 2011; Tang et al., 2018). Cell deformation itself little affects the whole volume but directly affects the tube diameter owing to changes in the shape of constituent cells. Active cellular rearrangement at the supra-cellular scale is also critical in regulating tube diameter (Andrew and Ewald, 2010; Walck-Shannon and Hardin, 2014; Rauzi, 2020).

## Examples of Tissue and Organ Size Regulation

In this section, two examples of feedback control systems for tissue and organ size regulation are described. These systems employ a homeostatic system in which individual cells sense and respond to mechanical forces.

### Maintenance of Epithelial Tissue Volume by Controlling Cell Number

The first example is the size regulation of monolayer epithelial tissues by increasing or decreasing the number of cells as the manipulated variable in the system. Contact inhibition of cell proliferation, wherein the proliferative ability of cells decreases under high-density conditions, was reported half a century ago (Levine et al., 1965). In 2005, Shraiman proposed a mechanical feedback control system that regulates the proliferative ability of cells by sensing the mechanical force received by cells from their surroundings (Shraiman, 2005). The main points proposed are as follows. When cells strongly pull each other under low-density conditions, intracellular tension increases, and cell proliferation is accelerated. In contrast, when cells play a weak tug-of-war or push each other under high-density conditions, cell proliferation is suppressed. Further, cell death is induced in response to the high pressure occurring in overcrowded conditions. This theoretical study has been followed by additional theoretical and experimental studies (Hufnagel et al., 2007; Aegerter-Wilmsen et al., 2012; Eder et al., 2017), leading to a comprehensive picture of the system, in which tissue size is regulated by manipulating the number of cells through cellular sensing and response to the mechanical forces in living tissues. A series of studies have attracted attention for elucidating the signaling molecules involved as regulators of this system.

One of the critical signaling pathways for tissue homeostasis as the controller in this system could be triggered by a mechanosensitive ion channel. Mechanical forces activate the Piezo1 when exerted on cellular membranes, triggering intracellular signal transduction through converting the mechanical stimuli (Coste et al., 2010; Saotome et al., 2018; Lin et al., 2019). It has been demonstrated that Piezo1 works as the sensor to monitor the deviation of mechanical states in epithelial tissues for the tissue volume homeostasis using epithelial cultured cell lines and the zebrafish. When epithelial cells are at a high density, Piezo1 is activated in response to compressive force loading on the cells, followed by the activation of sphingosine 1-phosphate and Rho-kinase-dependent pulsatile myosin contraction, resulting in the cell extrusion (Eisenhoffer et al., 2012; Atieh et al., 2021). Interestingly, Piezo1 is also activated at a low cell density. In this case, cells sense the existing tension and respond by activating extracellular signal-regulated kinases (ERKs) *via* an increase in intracellular calcium ion concentration, eventually leading to cell proliferation (Gudipaty et al., 2017). In other words, Piezo1 uses different downstream signaling pathways depending on the degree of mechanical forces experienced by the cells. Knockdown of Piezo1 or inhibition of ERK activity prevented stretch-induced mitosis, indicating that each factor takes a key role as a sensor and a controller in the feedback system (Gudipaty et al., 2017). However, the molecular mechanisms underlying mechanical feedback systems are not uniquely determined and further elucidation of the signaling mechanism is needed.

Recently, many efforts have been made to better understand how the mechanical and chemical changes in tissues are locally coordinated to eliminate cells for the homeostatic maintenance overall the epithelial tissues (Gudipaty and Rosenblatt, 2017; Ohsawa et al., 2018). Quantitative imaging approaches have revealed that the epithelial cells tend to be eliminated by compression-driven ERK inactivation (Moreno et al., 2019), and the extruding cells are governed through an interplay between actomyosin contractility and cell junctions (Lubkov and Bar-Sagi, 2014; Thomas et al., 2020). Moreover, the neighboring cell behaviors are also physically affected by the extruding cells through intercellular signal transmissions, such as calcium and ERK activation waves (Takeuchi et al., 2020; Gagliardi et al., 2021; Valon et al., 2021). These findings led to accelerating studies on mechanical cell competition – the mechanism for eliminating unfit cells for the tissue homeostasis (Brás-Pereira and Moreno, 2018; Matamoro-Vidal and Levayer, 2019).

### Maintenance of Epithelial Tube Diameter Through Cell Rearrangement

The second example is a mechanical feedback regulation to maintain tubule diameter, which is a physiologically important quantity of tissue structure. The epididymal tubule in the male reproductive tract is an experimental system that allows the examination of epithelial tube morphogenesis (Joseph et al., 2009; Murashima et al., 2015). During murine development, epithelial cells in the epididymal tubule divide in all directions on the tangential plane of the tubule (Xu et al., 2016; Hirashima and Adachi, 2019). As the cells divide longitudinally as well as along the circumferential axis of the tubule, the tubule diameter is expected to increase with time; however, the diameter of the epididymal tubules hardly changes throughout the morphogenetic process (Joseph et al., 2009; Hirashima, 2014). Feedback systems that have been poorly understood may be involved in controlling the deviation in the tube diameter from the target value.

**FIGURE 2:**
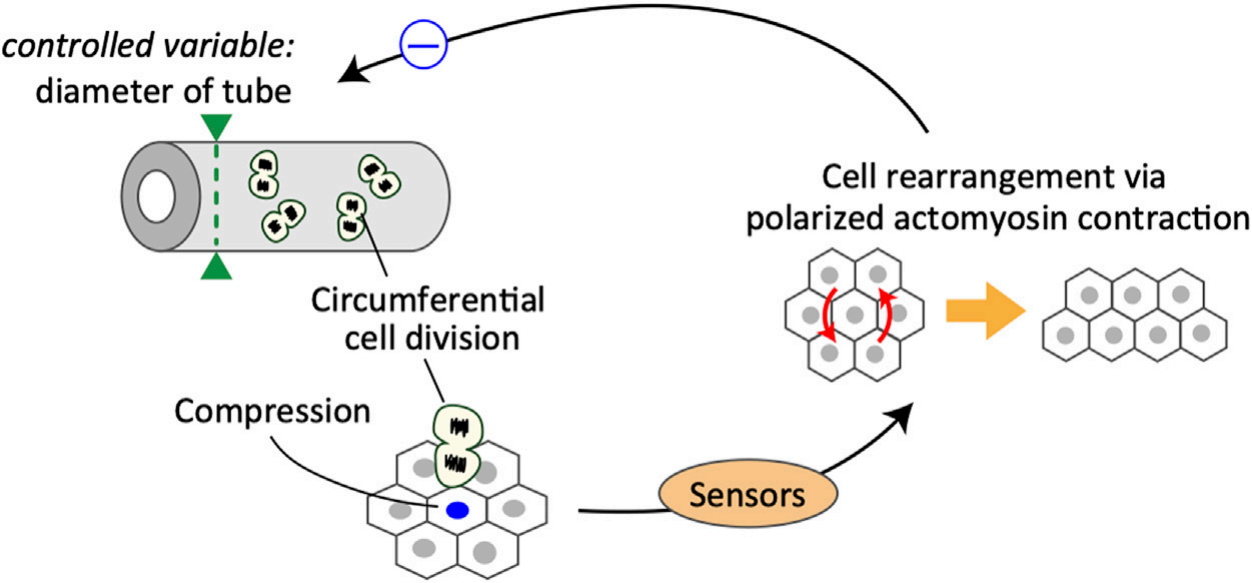
Schematics of a control system for the maintenance of epididymal tubule diameter.

The cellular dynamics of embryonic murine epididymal tubules under *ex vivo* culture conditions were examined using two-photon live-cell imaging. Live imaging analysis revealed that a group of cells adjacent to the dividing cells were more likely to cause cell rearrangement *via* actomyosin contraction in response to cell division along the tubule circumference (Hirashima and Adachi, 2019). Importantly, oriented cell division in the circumferential axis of tubules transmits mechanical signals through compressive forces, which would trigger polarized myosin activation to maintain the tube diameter (Figure 2). The obtained quantitative data were incorporated into a mathematical model of multicellular dynamics, and it was confirmed that the mechano-response system would maintain the diameter of developing tubes. Taken together, the analysis suggests that the polarized mechano-responsive cellular behavior at the supra-cellular scale maintains tube diameter at the whole-tissue scale (Hirashima and Adachi, 2019).

This is a typical example of a negative feedback control system, in which cells sense the increase in cell number along the circumferential axis of the tubules and the corresponding change in mechanical forces, eventually leading to active cell rearrangement for regulating the tube diameter (Figure 2). In this case, the controller in the system is partially composed of Rho-kinase-dependent acto-myosin contraction, and the manipulated variable is the cell position regulated by the cell rearrangement. One important but unclear aspect is the mechanism by which cells sense the size of a specific dimension. In the case of developing epididymal tubules, it is important to understand how epithelial cells acquire the information of specific orientation in sensing mechanical stimuli. One possible factor that provides information regarding the orientation of cells is planar cell polarity (PCP) proteins. PCP signaling activates Rho-associated kinase, an upstream kinase of non-muscle myosin (Nishimura et al., 2012; Guillot and Lecuit, 2013; Butler and Wallingford, 2017). Importantly, PCP proteins, such as Vang-like (VANGL) and tyrosine-protein kinase-like 7 (PTK7), are mainly localized on the apical junctions of tube cells circumferentially, and loss of PCP causes failure of cell arrangement, eventually leading to radial tube expansion in the epididymis (Xu et al., 2016; Hirashima and Adachi, 2019) and kidney (Karner et al., 2009; Kunimoto et al., 2017). Thus, PCP proteins likely serve as core regulators of the polarized mechano-response system. However, the sensors involved and their ability to function in an integrated manner remain poorly understood and should be examined further in future studies.

Epididymal cells possess the ability to sense compressive forces, specifically along the circumferential axis of proliferative tubules. In response to compressive forces, cells undergo oriented cell rearrangement by generating actomyosin-based polarized contractile forces, which eventually suppress the increase in tube diameter due to circumferential cell division.

## Conclusions and Discussion

This review describes the mechanical feedback systems for multicellular tissue size maintenance. First, a general framework of the feedback loop underlying tissue size control has been introduced with a few physiological and pathological examples. As building blocks of tissues, cells seem to possess inherent size regulation systems as in collectives. Considering this, mapping each component in the feedback control system to biological events allows us to capture the phenomena from different views by simplifying the system. These have been presented using two examples of tissue size regulation, i.e., maintenance of epithelial tissue volume by controlling cell number and maintenance of epithelial tube diameter through cell rearrangement. Throughout this minireview, I have discussed the cellular responses to mechanical forces involving collective multicellular behaviors for organizing tissue size control. Although the multicellular mechanoresponse is not a sole regulatory mechanism, this would be a principle of cell-to-cell communication through cellular sensing of and responses to mechanical forces for tissue size homeostasis. These mechanoresponsive cellular behaviors likely play a pivotal role in the systemic regulation. The existence of feedback systems, where each component is well-coupled for the tissue size regulation, is a premise of this minireview, and biological outcomes caused by partial defects in the sensor or controller raised in the two examples support it. However, it remains unclear how the feedback components organize as a whole system for the tissue size control. Further efforts, especially to identify the molecules involved in sensing mechanical forces, are anticipated.

I expect that the polarized cellular mechano-response systems introduced in *Maintenance of the Epithelial Tube Diameter Through Cell Rearrangement* Section would serve as a fundamental mechanism for tissue morphogenesis during development and growth. Individual cells should have their polarity along each axis of tissue coordinate. Accordingly, molecular machineries responsible to the mechano-sensing would be localized at a subcellular scale according to the cell polarity, causing subsequent chemical signaling responding to the mechanical stimulus against the specific orientation. Maintaining the size along the specific axis in growing tissues links to another aspect of anisotropic tissue morphogenesis, which gives rise to diverse tissue shapes. Despite its importance, experimental studies are currently limited. Provided that constituent cells are loosely connected to each other under a low-density condition, the change in cell tension due to neighbor cell divisions would be negligible and the mechanical forces may not work as a signal to control the tube diameter. In that case, identifying what signals control the tube diameter is also demanded. I hope that future studies will fill the gap in understanding the polarized mechano-response system.
